# Iodine Status After Bariatric Surgery—a Prospective 10-Year Report from the Swedish Obese Subjects (SOS) Study

**DOI:** 10.1007/s11695-017-2833-0

**Published:** 2017-08-02

**Authors:** Sofia Manousou, Lena M. S. Carlsson, Robert Eggertsen, Lena Hulthén, Peter Jacobson, Kerstin Landin-Wilhelmsen, Penelope Trimpou, Per-Arne Svensson, Helena Filipsson Nyström

**Affiliations:** 1 Department of Medicine at Kungälvs Hospital, Kungälv, Sweden; 2 Institute of Medicine, Sahlgrenska Academy, University of Gothenburg, Gothenburg, Sweden; 3 Department of Internal Medicine, Lasarettsgatan Kungälv’s Hospital, SE-442 34 Kungälv, Sweden; 4 Mölnlycke Health Care Center, Mölnlycke, Sweden; 5 Department of Internal Medicine and Clinical Nutrition, Sahlgrenska Academy, University of Gothenburg, Gothenburg, Sweden; 6 Department of Medicine, Section for Endocrinology, Sahlgrenska University Hospital, Gothenburg, Sweden

**Keywords:** Bariatric surgery, Gastric bypass, Vertical gastric banding, Iodine, Swedish Obese Subjects study, Obese

## Abstract

**Context:**

Bariatric surgery can lead to nutrient deficiencies. Gastric by-pass (GBP) entails restriction and malabsorption, whereas, vertical banded gastroplasty (VBG) is only restrictive.

**Objective:**

The objective of this study is to study whether GBP-patients develop iodine deficiency from malabsorption, and if GBP- and VBG-patients develop lower 24-h urinary iodine excretion (24-UIE) than obese non-operated controls (OB-controls) due to lower iodine intake.

**Design:**

The Swedish Obese Subjects (SOS) study is a prospective, non-randomized study of 4047 obese patients included 1987–2001, who chose bariatric surgery or non-surgical treatment. SOS-groups were compared at baseline, after 2 and 10 years and with population-based subsamples (MONICA-controls).

**Patients:**

One hundred eighty-eight GBP-patients were matched with 188 VBG-patients and 188 OB-controls and with three subgroups from 412 MONICA-controls.

**Main Outcome Measurements:**

Primary outcome was 24-UIE. Secondary outcomes were iodine intake, iodine supplementation, TSH, FT4, and thyroid morbidity.

**Results:**

At baseline, median 24-UIE was higher in GBP-patients, VBG-patients and OB-controls than in MONICA-controls (214, 201, 203 and 137 μg/day, *p* < 0.001). At 10 years, 24-UIE in GBP-patients (161 μg/day) and VBG-patients (149 μg/day) was lower compared with baseline (*p* < 0.01) and OB-controls (189 μg/day, *p* < 0.01), but similar to 24-UIE in MONICA-controls (137 μg/day). The 10-year-dietary iodine intake was similar in GPB-patients and OB-controls, but higher in VBG-patients. Iodine supplementation was taken by 0–9% in SOS-groups.

**Conclusion:**

After surgery, GBP- and VBG-patients did not suffer from iodine deficiency, but both groups had lower iodine status than OB-controls. Dietary supplements recommended after bariatric surgery do not need to include iodine, in iodine sufficient countries.

**Trial Registration:**

clinicaltrials.gov: NCT01479452

## Introduction

Bariatric surgery is increasingly used [1, 2] and can lead to several nutrient deficiencies [3]. The hypothesis on iodine deficiency after bariatric surgery was generated from a study in 1964 [4]. Bariatric surgery procedures are either restrictive, malabsorptive, or a combination thereof. Vertical banded gastroplasty (VBG) is a restrictive surgery, no longer performed, where a stomach pouch is created, but the intestine remains intact [5]. Gastric by-pass (GBP) is a widely performed combined technique, where a gastric pouch is created and both the ventricle and part of the small intestine are by-passed [5]. GBP is also malabsorptive and the patients are advised to take iron, B12, calcium and vitamin D supplementation [6, 7]. Regardless of the type of surgery, patients may have altered food preferences or vomiting problems [6, 8]; hence, both iodine intake and uptake may be influenced.

Iodine is important for the production of thyroid hormones [9–13]. In Sweden, the main iodine sources are iodized salt, dairy products, fish and seafood [14]. Before absorption, ingested iodine and iodate are converted to iodide in the gastrointestinal tract [15]; an iodine transporter in the small intestine seems to mediate recycling [16]. Iodine status in a population is commonly determined by urinary iodine concentration (UIC) in spot urine [17], with a reference range of UIC 100–199 μg/L [18]. 24-UIE is used as the best marker of iodine intake [19].

Since the study in 1964 [4], only two trials have investigated iodine status after bariatric surgery without confirming the iodine deficiency hypothesis; οne from Greece (*n* = 35) with a 6-month postoperative follow-up [20], and one from Spain (*n* = 90) with cross-sectional design [21]. Longer follow-up studies are lacking.

The aim of this study was to investigate a subsample of the large Swedish Obese Subjects (SOS) study [22] before, 2 and 10 years after bariatric surgery. The hypothesis was that patients that have undergone GBP develop iodine deficiency due to iodine malabsorption, regardless of dietary alterations after surgery, and that both GBP- and VBG-patients have lower 24-UIE than obese non-operated controls (OB-controls), due to lower iodine intake.

## Materials and Methods

### Study Design

The SOS study is a non-randomized, prospective study (*n* = 4047) with ongoing follow-up of obese patients recruited 1987–2001 [22]. Individuals who chose surgical treatment formed the surgery group (*n* = 2010) and a non-randomized contemporaneously matched control group (*n* = 2037) was created. In the iodine sub-study, data from the SOS study at 0, 2 years and/or 10 years after inclusion were collected. These data included body mass index (BMI), smoking, kidney function tests, urinary sodium (U-Na) as a marker of salt intake, dietary habits, use of vitamin supplements, urinary collection time, urinary volume, free thyroxin (FT4), thyrotropin (TSH), thyroid morbidity and medication. For the purposes of this sub-study, 24-UIC was analyzed and 24-UIE was calculated. The patients in the SOS-study were compared with a random population-based sample (*n* = 412) from the World Health Organization MONItoring of trends and determinants for CArdiovascular disease (WHO MONICA) Gothenburg, examined in 2008 [23]. Information on BMI, smoking, kidney function, U-Na, FT4, TSH, 24-UIC and 24-UIE was collected from the MONICA-database.

### Participants

**Fig. 1:**
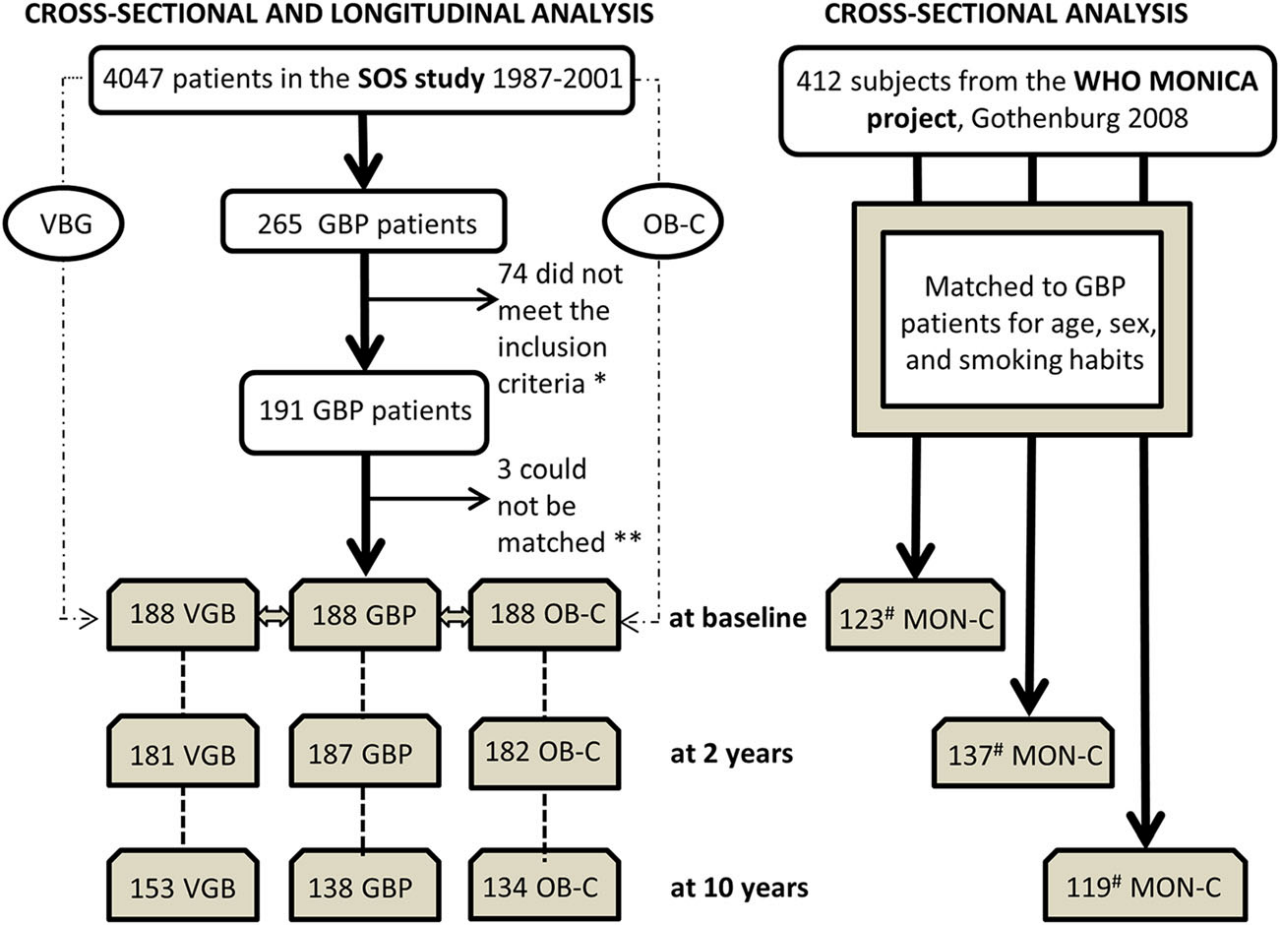
Flow chart of the recruitment of 564 patients from the Swedish Obese Subjects (SOS) study where equal groups of patients with vertical banded gastroplasty (VBG, *n* = 188) and obese non-operated controls (OB-C, *n* = 188) were matched with patients that had undergone gastric bypass (GBP, *n* = 188) surgery. The three SOS-groups have been followed for 10 years. Patients with GBP were also matched with controls from a random population-based sample, the World Health Organization MONItoring of trends and determinants for CArdiovascular disease, Gothenburg, Sweden, the WHO MONICA project (MON-C, *n* = 412). S*ingle asterisk*, 24-urine samples at 0, 2, and/or 10 years after inclusion. *Double asterisks*, GBP matched to VGB and OB-controls for age, sex, BMI, and smoking habits. *Number sign*, on each matching occasion—baseline, 2 and 10 years—the whole MONICA population (*n* = 412) was used

From the surgical group of the SOS-study (*n* = 2010), 265 patients had undergone GBP. The inclusion criteria for the GBP-group of this sub-study were availability of 24-h urine samples at 0, 2 and/or 10 years after recruitment and possibility to be matched to VBG-patients and OB-controls. Therefore, all participants in this sub-study had left 24-urine samples at baseline and at least once more. VBG-patients were selected from the surgical group of the SOS-study, whereas OB-controls were selected from the non-randomized contemporaneously matched control group of the SOS-study. Through matching the 2008 MONICA-population to the corresponding GBP-group at 0, 2 and 10 years, three MONICA-subpopulations were created (Fig. 1). Informed consent was obtained from all individual participants included in the study.

### Dietary Iodine Intake and Iodine Containing Supplements

These data were collected from food frequency questionnaires. Dietary iodine intake was calculated based on the assumption that one glass of milk contained 30 μg iodine, one plate of yogurt contained 40 μg, and one meal of fish contained 120 μg [24]. Patients with an iodine supplementation were defined as patients reporting taking multivitamins with iodine ≥50% of RDI (i.e., ≥75 μg/day). Patients with no iodine supplementation were defined as patients reporting taking multivitamins with iodine <50% of RDI (i.e., <75 μg/day) or no multivitamins.

### U-Na, U-Albumin, S-Creatinine, 24-UIC and 24-UIE

Twenty-four-hour urine samples were collected and U-Na, U-Albumin and S-Creatinine were measured according to the routine method at the accredited Laboratory of Clinical Chemistry at Sahlgrenska University Hospital, Gothenburg, Sweden. 24-UIC was measured blindly by an experienced lab engineer at the Section for Clinical Nutrition in Sahlgrenska Academy, University of Gothenburg, Gothenburg, Sweden, with the modified Sandell-Kolthoff reaction [25]. The method was validated through the EQUIP network and samples were measured in duplicate and reanalyzed, if difference in absorbance was >2%. Where the reported urine time collection was <20 or >28 h, appropriate mathematical adjustments were made. 24-UIC value was multiplied by the urine volume to calculate 24-UIE.

### FT4 and TSH Measurements, Frequency of Thyroid Diseases and Thyroid-Related Medication

Method description is given in the Appendix.

### Statistical Methods

Categorical variables are presented as *n* (%), and continuous variables are presented as mean and SD or median, Q1 and Q3. For pairwise group comparison, Fisher’s exact test was used for dichotomous variables and Mann-Whitney *U* test for continuous variables. Sign test was used for changes in dichotomous variables within a group and Wilcoxon sign rank test was used for changes in continuous variables. All statistical testing was at alpha significance of 0.05. All statistical analyses were performed with SAS software version 9.4 (SAS Institute Inc., Cary, NC, USA).

## Results

The sample size is presented in Fig. 1. Background information and data on iodine intake are presented in Table 1. Median iodine intake decreased from baseline to 10 years only in GBP-group (from 770 to 660 μg/week, *p* < 0.001).

**Table 1:** Baseline characteristics of patients within the Swedish Obese Subjects (SOS) study subdivided in those operated with gastric bypass (GBP), with vertical banded gastroplasty (VBG) and obese non-operated controls (OB-C). The obese patients were compared with controls from a random population based sample, WHO MONICA project, Gothenburg (MON-C). Data from 0 (baseline), 2 and 10 years follow-up are shown for sample size, smoking frequency, body mass index (BMI), U-Na, dietary iodine intake, and use of supplements

Years		GBP	VBG	OB-C	MON-C	Test between groups, *p* value					
						GBP VBG	GBP OB-C	GBP MON-C	VBG OB-C	VBG MON-C	OB-C MON-C
Sample size	0	*n* = 188	*n* = 188	*n* = 188	*n* = 123						
	2	*n* = 187	*n* = 181	*n* = 182	*n* = 137						
	10	*n* = 138	*n* = 153	*n* = 134	*n* = 119						
Sex—female *n* (%)	0	141 (75.0)	141 (75.0)	141 (75.0)	138 (74.6)	ns	ns	ns	ns	ns	ns
Age (year) mean (SD)	0	47.4 (6.0)	47.5 (5.8)	49.0 (6.0)	54.2 (6.8)	ns	0.013	<0.001	0.015	<0.001	<0.001
BMI (kg/m^2^) mean (SD)	0	43.7 (4.3)	43.3 (4.4)	42.0 (4.1)	25.7 (3.6)	ns	<0.001	<0.001	<0.001 <	<0.001	<0.001
	2	30.0 (4.1)	33.3 (4.6)	41.4 (4.5)	25.8 (3.8)	<0.001	<0.001	<0.001	<0.001	<0.001	<0.001
	10	33.2 (5.2)	36.2 (5.2)	41.8 (5.4)	26.5 (4.3)	<0.001	<0.001	<0.001	<0.001	<0.001	<0.001
Smokers *n* (%)	0	36 (19.1)	36 (19.1)	36 (19.1)	16 (13.0)	ns	ns	ns	ns	ns	ns
	2	35 (19.2)	39 (21.8)	32 (17.9)	15 (10.9)	ns	ns	0.060^§^	ns	0.015	ns
	10	32 (23.2)	30 (20.4)	17 (12.4)	23 (19.3)	ns	0.029	ns	0.097^§^	ns	ns
S-Creatinine mmol/l mean (SD)	0	87.8 (11.3)	86.4 (10.0)	86.7 (10.7)	69.9(12.3)	ns	ns	<0.001	ns	<0.001	<0.001
U-Albumin mg/mmol median (IQR)	0	7.9 (4.3;16.0)	9.2 (4.5;18.0)	7.6 (4.2;16.8)	–	ns	ns	–	ns	–	–
U-Na mmol/day mean (SD)	0	127 (44.3)	121 (44.0)	118 (46.9)	98 (47.9)	ns	0.051^§^	<0.001	ns	<0.001	<0.001
	2	112 (42.4)	106 (44.5)	112 (45.6)	97 (46.9)	ns	ns	<0.001	ns	0.034^§^	0.001
	10	110 (44.3)	107 (42.4)	113 (40.7)	94 (43.1)	ns	ns	0.002	ns	0.004	<0.001
Dietary iodine intake^a^ μg/week median (IQR)	0	770 (515;1270)	870 (570;1290)	730 (480;980)	–	ns	ns	–	0.012	–	–
	2	685 (450;940)	890 (570;1250)	735 (520;1040)	–	<0.001	ns	–	0.006	–	–
	10	660 (390;870)	900 (540;1230)	700 (450;960)	–	<0.001	ns	–	0.002	–	–
Change in dietary iodine intake from 0 to 10 years (μg/week) median (IQR)		−135.0 (−490;120)	0.00 (−210;290)	−95.0 (−230;180)	–	<0.001	0.036	–	ns	–	–
Intake of any supplement *n* (%)	0	44 (23.4)	43 (22.9)	52 (27.7)	–						
	2	80 (42.7)	48 (26.5)	57 (31.3)	–						
	10	80 (58.0)	54 (35.3)	62 (46.3)	–						
Intake of iodine containing supplement *n* (%)	0	3 (1.6)	0 (0.0)	3 (1.6)	–						
	2	14 (7.5)	10 (5.5)	3 (1.6)	–						
	10	12 (8.7)	14 (9.2)	5 (3.7)	–						
No iodine supplement intake^b^ *n* (%)	0	184 (97.9)	184 (97.8)	179 (95.2)	–						
	2	161 (86.1)	162 (89.5)	177 (97.3)	–						
	10	123 (89.1)	135 (88.2)	125 (93.3)	–						
Intake of supplement with uncertain content *n* (%)	0	1 (0.5)	4 (2.1)	6 (3.2)	–						
	2	12 (6.4)	9 (5.0)	2 (1.1)	–						
	10	3 (2.2)	4 (2.6)	4 (3.0)	–						

*ns* non-significant (*p* ≥ 0.10)

^§^Marginally significant (0.05 < *p* < 0.10)

^a^Assessment based on milk, yogurt and fish digestion, from patient questionnaires

^b^No iodine supplement intake group: those not taking any supplement together with those taking supplement without iodine

### 24-UIC and 24-UIE

**Fig. 2:**
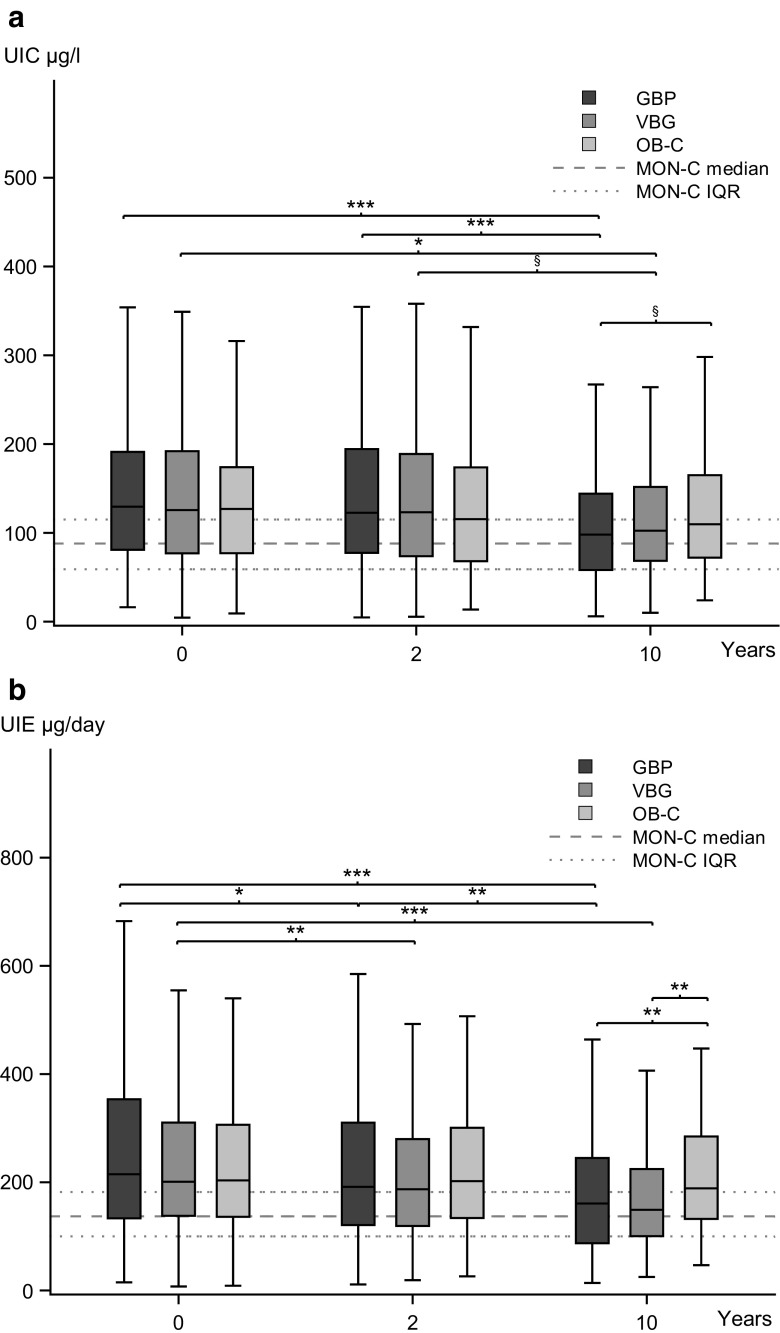
Box plot showing the distribution of 24-h urine iodine concentration (24-UIC) (**a**) and 24-h urine iodine excretion (24-UIE) (**b**) of the gastric bypass (GBP), vertical banded gastroplasty (VBG), and obese non-operated (OB-C) group at baseline, and 2 and 10 years after inclusion, and median and interquartile range of the three subgroups from the WHO MONICA project (MON-C). *P* values from longitudinal and cross-sectional analyses of the three obese patient groups are presented. ***p < 0.001, **p < 0.01, *p < 0.05, ^§^ *p* < 0.10

In the three groups of SOS-patients, baseline median 24-UIC was similar and within the reference range, i.e., within 100–199 μg/L, but it was higher than in MONICA-controls (*p* < 0.001). 24-UIC decreased from baseline to 10 years in GBP- and VBG-groups but was still within the reference range; whereas, it was unchanged in OB-controls. At 10 years, 24-UIC was similar in the GBP-patients and MONICA-controls; whereas, it was still higher in the VGB-patients than in MONICA-controls (*p* = 0.002) (Fig. 2a).

Baseline median 24-UIE exceeded 200 μg/day in all three SOS-groups, which was higher than the 137 μg/day observed in MONICA-group (*p* < 0.001). From baseline to 10 years, 24-UIE decreased similarly in both the GBP- (215 to 161 μg/day) and VBG-group (201 to 149 μg/day) and was similar to MONICA-group (137 μg/day) at 10 years. However, in OB-controls, 24-UIE was unaltered (203 at baseline and 189 μg/day at 10 years) (Fig. 2b).

After exclusion of the few SOS-patients taking supplements with iodine or with uncertain content (Table 1), the results for 24-UIC and 24-UIE were unaltered. A sensitivity analysis of those with urine samples on all three occasions compared to the analysis of the whole groups provided similar results. The SOS-patients taking iodine containing supplement were too few (Table 1) for statistical analysis.

### FT4, TSH, Thyroid Morbidity and Medication

Iodine deficiency was not found in the operated groups. Hence, the statistical analysis of thyroid function tests lacked clinical relevance; data are presented in Appendix.

## Discussion

The results did not support the hypothesis that GBP patients in Sweden suffer from iodine deficiency, but supported the hypothesis that both GBP- and VBG-patients have lower iodine status after surgery, compared with the non-operated obese counterparts (OB-controls); the contribution of dietary alterations and malabsorption to this reduction are indeterminate. The three SOS-groups (GBP-patients, VBG-patients and OB-controls) had similar 24-UIE at baseline, which was higher than in the random population-based sample (MONICA-controls). Both GBP- and VBG-groups had similar 24-UIE to the MONICA-group at 10 years. This effect was similar irrespective of surgical methods (GBP or VBG), indicating no iodine malabsorption specifically after GBP.

This study is to our knowledge the largest and longest prospective study on iodine status after bariatric surgery. The only longitudinal study, to date, is from Greece [20], and comprised 35 patients before and up to 6 months after surgery: this study reported normal UIE at baseline and at 6 months, with a transient iodine excess at 3 months. The decrease in UIE, seen in our study, was not observed in the Greek study, possibly because of the shorter follow-up and the small sample size. Cohorts of less than 100 subjects are probably too small for a safe evaluation of UIE, due to large intra-individual variation [26]. A cross-sectional study from Spain [21] evaluated 90 women 1.5–5 years after bariatric surgery and compared these women with 45 non-obese controls and 90 non-operated obese women. That study reported normal UIC in spot urine (expressed as iodine-to-creatinine ratio) in operated women, which was lower than in non-obese controls; however, the study also reported iodine deficiency in non-operated obese subjects. Therefore, the Spanish study suggested obesity infers iodine deficiency and that surgery promotes normalization of UIC, which contrasted with the results from the present study. Several differences between the studies could explain the contradictory results, for instance the cross-sectional design and the absence of a BMI-matched group to the post-bariatric patients. Many obese non-operated patients followed weight reducing regimes, which could explain the iodine deficiency in obesity, a situation with generally high dietary energy intake [27]. 24-UIE was measured in the present study, which is a more accurate marker than spot UIC with iodine-to-creatinine ratio [19]. However, in both the Greek [20] and the Spanish [21] studies, iodine status was normal after BS, which was in agreement with the results presented in this study.

The normal iodine status after bariatric surgery in this study was contrary to the study from 1964 [4], where 2.5–12 years after surgery, six of the eight patients with total gastrectomy and esophageal jejunal anastomosis had subnormal 24-UIE. Mean 24-UIE in that study was 46 μg/day in the operated patients and 90 μg/day in the controls (*p* < 0.01), despite an estimated iodine intake at 200% RDI, indicating iodine malabsorption. However, as the present study was longitudinal and concerned modern surgical techniques with obviously larger remaining gastrointestinal tract, a comparison with the study from 1964 is inappropriate.

The current data did not indicate lower iodine intake in the operated groups, compared with OB-controls at 10 years, although this was the most plausible explanation for the observed decrease in 24-UIE after surgery. The intake of iodine-rich products, registered in patient questionnaires, was similar in GBP-patients and OB-controls on all occasions, whereas, the VBG-group had the highest iodine intake at both baseline and 10 years. Longitudinal data indicated only the GBP-group altered their iodine intake after surgery; even so, this was only a decrease of 110 μg/week, i.e. 16 μg/day. U-Na was also analyzed as a proxy for the total salt intake, given that most table salt is supplemented with iodine. U-Na was similar in all SOS-groups on all occasions and lower in MONICA-groups. This was consistent with more ingested food among obese patients, and indicated a tendency to use the same amount of salt both before and after surgery.

U-Na and the registered dietary iodine intake have several limitations as proxies for the total iodine intake. It was impossible to know the amount of iodine in ingested salt. Iodinated salt is used in some products, but the food industry tends to use cheaper and more feasible non-iodinated salt. In addition, the use of dietary questionnaires generate a recall bias, and there is a tendency for obese patients to under-report food intake [28]. However, these factors should have affected the SOS-groups similarly and thereby should not have induced a dependent measurement error in the analyses. Another limitation was the questionnaires were not primarily designed to study iodine intake. As bariatric surgery may affect food preferences [29], the three SOS-groups at baseline and the OB-controls at 10 years may have eaten non-registered iodine-rich food, which operated patients may have become reluctant to consume.

Another possible explanation for the lower 24-UIE after surgery in presence of unaltered iodine intake was that both the GBP- and VBG-groups suffered from iodine malabsorption, which was masked by excessive iodine intake. Several factors may influence iodine absorption: (i) the removal of the part of the gastrointestinal tract where iodine and iodate are converted to absorbable iodide; (ii) the lack of digestion normally occurring in the stomach; (iii) the lack of recycling of iodine; and (iv) a possible rapid emptying of the stomach. Even though factors (i–iii) concern only GBP-patients, factor (iv) affects both GBP- and VBG-patients and could explain the similar 24-UIE decrease in the operated groups. Therefore, further research is needed on the physiology of iodine absorption and its alterations after bariatric surgery.

Even though this was the largest and longest study on iodine after bariatric surgery, there were some limitations. Besides the weaknesses in the collection of dietary data, it was a non-randomized study with the risk of residual confounding by variables not measured. Sample size was prioritized above a complete match between groups; however, adjustment for baseline differences in BMI and age rendered unchanged effect size and there were no group differences in gender, renal function, and smoking habits. The decision to include patients with urine samples available at baseline and 2 and/or 10 years after bariatric surgery may induce selection bias, as those with more gastrointestinal symptoms, and therefore, worse iodine status, may have refrained from monitoring; conversely, they may have been more motivated towards monitoring. Furthermore, a sensitivity analysis of those with urine samples on all three occasions compared to the analysis of the whole groups provided similar results. An important methodological strength was the primary endpoint of 24-UIE, which is a more reliable group marker for iodine intake than estimated UIE in spot urine, and that all measurements were done by the same experienced laboratory engineer. Other strengths included the large sample size and the comparison with a random population-based control-group.

## Conclusions

Bariatric surgery in iodine sufficient countries does not result in iodine deficiency, even though it decreases iodine levels. Hence, the hypothesis of this study was disproved. We could not explain the decreased iodine levels by our estimates of iodine intake or altered absorption. Dietary supplements recommended after bariatric surgery do not need to include iodine, in iodine sufficient countries.

## Appendix

### FT4 and TSH Measurements, Frequency of Thyroid Diseases and Thyroid-Related Medication

Fasting serum samples were drawn, centrifuged and transported unfrozen to a central lab within 24 h. The analysis of FT4 and TSH was done in connection to each visit. During the study, the Department of Clinical Chemistry, Sahlgrenska University Hospital, Göteborg, Sweden, used different methods: Kodak Amerlite (KodakClinical Diagnostics Ltd., Amersham, UK) from 1987 until November 1998; Vitros Amerlite (J&J Ortho-Clinical Diagnostics, Amersham, UK) until May 2000; Abbott Architect (Abbott Park, IL, USA) until May/June 2005; thereafter, Roche Modular (Roche Diagnostics, Mannheim, Germany). To improve the comparison between these analyses with different reference ranges, TSH and FT4 were only analyzed cross-sectionally at baseline and after 10 years, with the dominant method on each occasion as reference. The results from the other methods were converted to the dominant method through correction factors obtained by the laboratory for cross evaluating a change in methodology. At baseline, 75% of the samples were analyzed by Kodak Amerlite, and at 10 years follow-up, 52% were analyzed by Abbott Architect (reference ranges in Table 2). Information on medication and thyroid disease during the first 10 years after inclusion and current medication were obtained through patient questionnaires. The frequency of overt and subclinical hyper- and hypo-thyroidism, based on FT4 and TSH results, was calculated in relation to the reference range.

**Table 2:** Free thyroxine (FT4), thyrotropin (TSH) and frequency of thyroid diseases (number (%)) are presented for patients operated with gastric bypass (GBP), vertical banded gastroplasty (VBG), obese non-operated controls (OB-C) and controls from a random population based sample, WHO MONICA project, Gothenburg (MON-C). *P* values from a cross-sectional comparison for baseline and 10 years follow-up are presented. Laboratory methods changed during follow-up, making longitudinal comparison impossible. Means (SD) are given

Analysis at baseline All methods used converted to Kodak Amerlite. Reference Range FT4 11.7–28.0 pmol/L, TSH 0.1–3.0 mUI/L										
	GBP	VBG	OB-C	MON-C	Test between groups, *p* value					
					GBP VBG	GBP OB-C	GBP MNC-C	VBG OB-C	VBG MNC-C	OB-C MNC-C
FT4 (pmol/L)	15.1 (4.1) *n* = 187	16.1 (3.6) *n* = 188	15.6 (3.9) *n* = 187	16.5 (3.5) *n* = 122	0.0032	ns	<0.001	ns	ns	0.004
TSH (mUI/L)	1.23 (0.84) *n* = 185	1.79 (1.31) *n* = 188	1.83 (2.20) *n* = 187	2.18 (1.38) *n* = 122	<0.001	<0.001	<0.001	ns	<0.001	<0.001
Overt hyperthyroidism	0	0	1 (0.5)	0	ns	ns	ns	ns	ns	ns
Subclinical hyperthyroidism	4 (2.1)	3 (1.6)	0	0	ns	ns	ns	ns	ns	ns
Overt hypothyroidism	2 (1.1)	3 (1.6)	6 (3.2)	0	ns	ns	ns	ns	ns	ns
Subclinical hypothyroidism	6 (3.2)	18 (9.6)	15 (8.0)	0	0.019	0.070^§^	ns	ns	ns	ns
Analysis at 10 years All methods used converted to Abbott Architect. Reference Range FT4 9–22 pmol/L, TSH 0.2–4.0 mUI/L										
	GBP	VBG	OB-C	MNC-C	Test between groups, *p*-value					
					GBP VBG	GBP OB-C	GBP MNC-C	VBG OB-C	VBG MNC-C	OB-C MNC-C
FT4 (pmol/L)	15.3 (2.2) *n* = 136	15.9 (2.8) *n* = 152	15.5 (2.4) *n* = 135	16.6 (3.6) *n* = 118	0.083^§^	ns	<0.001	ns	0.039	0.003
TSH (mUI/L)	2.29 (1.65) *n* = 136	2.03 (1.47) *n* = 151	2.48 (1.29) *n* = 135	2.44 (1.89) *n* = 118	ns	0.020	ns	<0.001	0.006	ns
Overt hyperthyroidism	0	4 (2.1)	0	3 (1.6)	ns	ns	ns	ns	ns	ns
Subclinical hyperthyroidism	1 (0.5)	1 (0.5)	1 (0.5)	4 (2.2)	ns	ns	ns	ns	ns	ns
Overt hypothyroidism	4 (2.1)	0	0	1 (0.5)	ns	ns	ns	ns	ns	ns
Subclinical hypothyroidism	9 (4.8)	12 (6.4)	13 (6.9)	8 (4.3)	ns	ns	ns	ns	ns	ns

Overt hyperthyroidism: TSH below the normal range and FT4 above the normal range; subclinical hyperthyroidism: TSH below the normal range and normal FT4; overt hypothyroidism: TSH above the normal range and FT4 below the normal range; subclinical hypothyroidism: TSH above the normal range and normal FT4

*ns* non-significant (*p* ≥ 0.10)

^§^Marginally significant (0.05 < *p* < 0.10)

### Results from the Comparison of FT4, TSH, Thyroid Morbidity and Medication

The differences between groups regarding FT4, TSH and thyroid morbidity, as calculated by these hormones, are presented in Table 2. Data from patient questionnaires on thyroid morbidity and medication showed no differences between the groups, with the exception of higher thyroxine prescription in all SOS-groups compared to MONICA-groups and in GBP-patients compared to VBG-patients (data not shown).
